# Clustering of serotypes in a longitudinal study of *Streptococcus pneumoniae *carriage in three day care centres

**DOI:** 10.1186/1471-2334-8-173

**Published:** 2008-12-30

**Authors:** Tuija Leino, Fabian Hoti, Ritva Syrjänen, Antti Tanskanen, Kari Auranen

**Affiliations:** 1 National Public Health Institute (KTL), Mannerheimintie 166, FIN-00300 Helsinki, Finland

## Abstract

**Background:**

*Streptococcus pneumoniae *(pneumococcus) causes a wide range of clinical manifestations that together constitute a major burden of disease worldwide. The main route of pneumococcal transmission is through asymptomatic colonisation of the nasopharynx. Studies of transmission are currently of general interest because of the impact of the new conjugate-polysaccharide vaccines on nasopharyngeal colonisation (carriage). Here we report the first longitudinal study of pneumococcal carriage that records serotype specific exposure to pneumococci simultaneously within the two most important mixing groups, families and day care facilities.

**Methods:**

We followed attendees (N = 59) with their family members (N = 117) and the employees (N = 37) in three Finnish day care centres for 9 months with monthly sampling of nasopharyngeal carriage. Pneumococci were cultured, identified and serotyped by standard methods.

**Results:**

Children in day care constitute a core group of pneumococcal carriage: of the 36 acquisitions of carriage with documented exposure to homologous pneumococci, the attendee had been exposed in her/his day care centre in 35 cases and in the family in 9 cases. Day care children introduce pneumococci to the family: 66% of acquisitions of a new serotype in a family were associated with simultaneous or previous carriage of the same type in the child attending day care. Consequently, pneumococcal transmission was found to take place as micro-epidemics driven by the day care centres. Each of the three day care centres was dominated by a serotype of its own, accounting for 100% of the isolates of that serotype among all samples from the day care attendees.

**Conclusion:**

The transmission of pneumococci is more intense within than across clusters defined by day care facilities. The ensuing micro-epidemic behaviour enhances pneumococcal transmission.

## Background

Contrary to most other pathogens, *Streptococcus pneumoniae *(pneumococcus) is not primarily spread by individuals with clinical disease but by healthy carriers who harbour the bacteria in the nasopharynx. Data about cases of pneumococcal disease are therefore of little use in studies of pneumococcal transmission and preventive interventions targeting transmission. Studies of transmission are currently of specific interest because new pneumococcal polysaccharide-conjugate vaccines affect not only pneumococcal disease but also nasopharyngeal carriage and through this have indirect population level effects on invasive disease [1]. Detailed data about carriage and transmission are therefore of utmost value when aiming to forecast the entire prevention potential of the conjugate vaccines.

Acquisition of pneumococci in children has previously been studied with regard to exposure to pneumococci within the family, the most immediate "mixing group" for pneumococcal transmission [2-6]. Both the extent of exposure (number of carriers) as in large families and closeness of contact as in crowded households enhance transmission and exert a higher force of infection on a child. On one hand, older siblings introduce pneumococci to their younger siblings [7-9]. On the other hand, newborn infants may enhance pneumococcal transmission in the family by simply increasing chances of mutual transmission within the family [10]. Family studies allow assessment of differences in susceptibility to acquiring carriage by controlling exposure to pneumococci and have revealed higher susceptibility to acquiring carriage in young children [11-13]. This, together with longer carriage in children [14,15]emphasizes the important role of young children in maintaining circulation of pneumococci.

In day care facilities children have both frequent and close contacts to transmit pneumococci to each other. Day care attendance has commonly been mentioned as one of major risk factors for pneumococcal carriage [7,16-19]. However, we have previously shown that when simultaneous carriage by family members was measured, the attendance to day care, as a proxy for exposure in day care, was not associated with risk of carriage in young Finnish children [10,11]. We now believe that this lack of association was due to the non-specificity of day care attendance as a measure of exposure without carriage data from day care groups in comparison to explicit knowledge of simultaneous carriage within the family.

To assess the relative importance of the two contact groups, family and day care, or the combined effect of these two groups simultaneously, detailed data about exposure to pneumococci are needed from both. We here report results from a study that was designed to collect exposure information from both mixing groups, families and day care facilities, on equal footing. The study was designed as a longitudinal follow-up of 3 day care centres where children, their families and the day care employees were invited to participate. Monthly nasopharyngeal swabs with epidemiological background data including contact information were collected for 9 consecutive months. We discuss the implication of the core-group mixing to pneumococcal epidemiology and post-vaccination predictions.

## Methods

### Study population and participants

All attendees with their family members and the employees in three day care centres (DCCs), Tommila, Kuusimäki and Salorinne, in three suburban communities near the city of Tampere, Finland, were invited to participate in the study. Altogether 213 individuals, of which 59 day care attendees, 31 siblings, 86 adult family members and 37 employees, were enrolled as study participants (Table 1). The mean age of the attendees at the enrolment was 4.2 years, and 8.2, 36 and 39 years for siblings, adult family members and employees, respectively. The attendees, hereafter referred to as index children, belonged to 20 families out of 30 families using the day care centre Tommila, 13 out of 50 in Kuusimäki, and 12 out of 38 in Salorinne. These numbers account for 74%, 30% and 59% of all children attending the three DCC's, respectively. The typical size of the 45 participating families was 4 (in 30/45 families), the range being from 3 to 9. Eight participating families had at least 3 children and five families had only 1 child.

**Table 1:** The number of participants in the three day care cohorts.

*Status (Mean age, years)*	*Number of participants*			
	Tommila (20 families)	Kuusimäki (13 families)	Salorinne (12 families)	**Total**

Index children (4.2)	25	17	17	**59**
Siblings (8.2)	12	8	11	**31**
Adult family members (35.5)	40	22	24	**86**
Day care employees (38.5)	11	18	8	**37**

Total	**88**	**65**	**60**	**213**

The participants are categorised as index children (i.e, day care attendees), siblings under 18 years of age, adult family members (all > 18 years of age, mostly parents) and day care employees. The age was determined at the enrolment

There were two siblings who started attending the day care during the follow-up. Four children stopped attending the day care. For simplicity of analysis, the status of these children was fixed to that at the first visit. Only one child who was sampled for 4 times had been vaccinated against pneumococcus.

The participating families represent a range of social backgrounds, typical for small town housing areas. The population in Finland is white Caucasian, and only Finnish speaking families were recruited. An informed consent was obtained from the adult participants and the parents of the child.

An additional cohort of 175 children from 8 different classes of 8–12 year olds in a nearby school of DCC Kuusimäki was invited to participate in a cross-sectional study. A total of 170 of the 175 invited children were enrolled. Informed consents were given by the children and their parents.

### Samples

Families and DCCs were followed for nine months. During the follow-up between September 2001 and May 2002, deep nasopharyngeal swabs (NP) were collected from the study participants at 10 monthly visits. Samples were collected with a calcium alginate swab [20]. For most of the 213 participants, the data are almost complete: 87% of the individuals have 9 or 10 nasopharyngeal samples. In 38 out of the 45 participating families all members were enrolled, in 4 families 1 member and in 3 families 2 members were not enrolled. Altogether 1941 samples were taken.

According to the study protocol the sampling for each individual was scheduled with 1 month intervals, starting from the first sampling. For one sampling round within a single DCC and the families linked to it, all samples were collected within ± 2 weeks. These time windows are denoted as visits in the sequel. Most samples (94%) were collected within ± 1 week. The school cohort samples were collected on April 16–25, 2002.

Pneumococci were cultured using methods recommended by the WHO trialists' network and identified and serotyped by standard methods [20]. Briefly, the NP samples were transported within 8 hours at +4°C in a tube with STGG medium to be stored at -85°C and sent later on dry ice to the bacteriological laboratory. Samples were cultured on selective plates (5 μg for each) and incubated for 18–24 hours at +37°C with 5% CO_2_. Pneumococci were identified and one representative colony was selected for serotyping using antisera from Statens Seruminstitut, Copenhagen. Only isolates of serogroups 6, 9, 18, 19, 23, 11, 15 and 35 were subtyped. In the analysis, serotypes 15B and 15C are considered as one type (15B/C) because of reversible serotype switching between them [21].

### Definitions and statistical methods

Serotype specific *acquisition of carriage *in an individual was defined as any instance of observed carriage of the serotype in question (*the target type*) after a sample negative for that type in the individual. *Family acquisition *was any instance of observed carriage of the serotype in any of the family members, thus referred to as an *introductory carrier *or, in the case of several simultaneous carriers, as *co-introductory carriers*, after absence of that serotype in the family at the previous visit. In the analyses of acquisition, only pairs of samples from two consecutive visits one month apart were considered. *Exposure *to pneumococci was defined in terms of carriage of the target type by at least one other individual in the mixing group at the previous visit one month earlier, with categories "no observed carriage", "carriage in the family only", "carriage in the DCC only", or "carriage in both". The individual was at risk of acquiring of a serotype when not carrying that type at the previous visit one month earlier. The distribution of acquired serotypes, i.e. among episodes of carriage, was similar to that based on all samples pooled together. For simplicity, we report the latter. In epidemiological terms, the relevant interpretation of the pooled sample pertains to the net-exposure to pneumococci. *A day care cohort *comprises all individuals in a DCC and the families linked to it.

A Poisson regression model was used to estimate the rates of pneumococcal acquisition relative to the category of "no observed carriage". Specifically, separate relative rates were specified for categories "carriage in the family only" and "carriage in the DCC only" and a multiplicative relative rate for "carriage in both". The rates were assumed common across all serotypes. The rates and their 90% confidence intervals (CI) were estimated using the R software.

## Results

### Individual level

Out of the 1941 samples, 10.5% (203) were positive for pneumococci. The frequency of carriage was dependent on age: 25.3% of samples (132/521) collected from the index children were positive for pneumococci whereas the proportion of positive samples in the siblings, mostly older than the index children, was 12.5% (36/287). The frequency of carriage was low in the adult family members (3.2%; 25/789) and similarly low in the day care employees (2.9%;10/344). Of the index children 80% (47/59) carried pneumococci at least once during the follow-up, and at least two pneumococcal acquisitions occurred in 57% of these children (27/47). Ten children carried three times, five carried 4 times and one child carried 6 times.

Table 2 presents the observed serotype distribution in the isolates, stratified according to the status of the individual.

**Table 2:** The proportion of isolates belonging to each serotype according to the status of the individual.

*Serotype*	*Index children (N, %)*	*Siblings (N, %)*	*Adult family members and day care employees (N, %)*	Total **(N, %)**
18C	20 (15.2)	8 (22.2)	1 (2.9)	**29 (14.3)**
9V	18 (13.6)	1 (2.8)	6 (17.1)	**25 (12.3)**
3	21 (15.9)	1 (2.8)	1 (2.9)	**23 (11.3)**
19F	13 (9.8)	6 (16.7)	3 (8.6)	**22 (10.8)**
15B/C	14 (10.6)	5 (13.9)	2 (5.7)	**21 (10.3)**
11A	9 (6.8)	6 (16.7)	1 (2.9)	**16 (7.9)**
35F	7 (5.3)	1 (2.8)	3 (8.6)	**11 (5.4)**
19A	8 (6.1)	0 (0.0)	1 (2.9)	**9 (4.4)**
6B	1 (0.8)	5 (13.9)	2 (5.7)	**8 (3.9)**
14	4 (3.0)	0 (0.0)	4 (11.4)	**8 (3.9)**
22	5 (3.8)	0 (0.0)	2 (5.7)	**7 (3.4)**
Others*	12 (9.1)	3 (8.3)	0 (0.0)	**15 (7.4)**
Non-typables	0 (0.0)	0 (0.0)	9 (25.7)	**9 (4.4)**

Total	**132 (100%)**	**36(100%)**	**35 (100%)**	**203 (100%)**

The isolates originate from the main study ie. from the day care cohorts.

* Other types (total number of isolates): 33 (3), 38 (3), 6A (2), 9N (2), 10 (1), 16 (1), 18B (1), 35B (1) and 7 (1).

### Day care centre level

Pneumococci were prevalent in all three day care centres (DCC). The proportion of positive samples in the index children was on average 25%, ranging from 9 to 56% at different visits per day care. At 28 of the 30 visits to the three DCCs, at least three different serotypes were detected in the day care cohort (DCC and the families linked to it). Altogether 8 to 13 different serotypes were observed in the index children of a single DCC during the whole follow-up, indicating a continuous turnover of serotypes.

**Figure 1:**
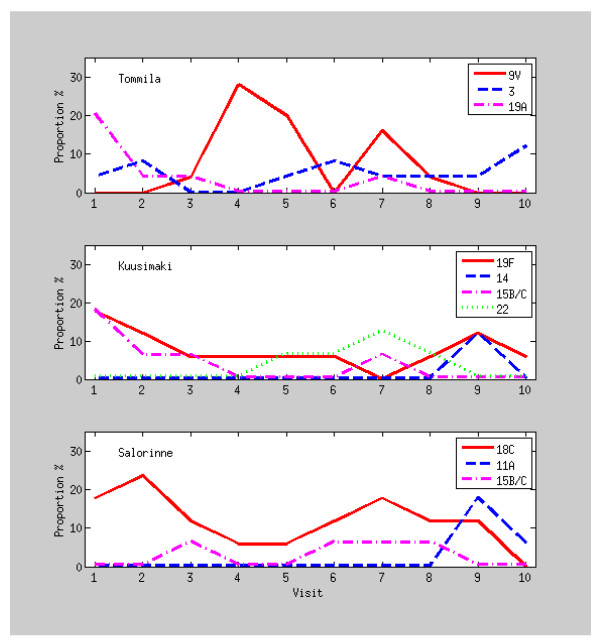
**The observed proportion of sampled index children carrying the most common serotypes in each day care centre by visit**.

Figure 1 presents the observed proportions of sampled index children carrying the most common serotypes in each DCC by visit. Each DCC was dominated by a serotype of its own: 9V in DCC Tommila, 19F in DCC Kuusimäki and 18C in DCC Salorinne (Table 3). In fact, all 9V isolates taken from index children were confined to DCC Tommila where they comprised almost a third of isolates from the index children (18/57). Correspondingly, all 19F isolates from index children were found in DCC Kuusimäki (36% of 36 isolates) and all 18C isolates in DCC Salorinne where they comprised more than half of the isolates (20/39 isolates). All the three dominant serotypes persisted in the day care centres practically through the whole follow-up time (Figure 1). Similarly to serotype 9V, serotype 19A were found only in DCC Tommila. Although the number of isolates (8 cases) is much smaller than that of 9V (18 cases), the cases were highly concentrated to the beginning of the study period.

**Table 3:** The distribution of serotypes among the pneumococcal isolates in the three day care centres.

	***Index children (N, %)***			All individuals (N, %)		
**Serotype**	**Tommila**	**Kuusimäki**	**Salorinne**	**Tommila**	**Kuusimäki**	**Salorinne**
18C	0	0	20 (51.3)	0	0	29 (43.3)
9V	18 (31.6)	0	0	25 (35.7)	0	0
3	12 (21.1)	2 (5.6)	7 (17.9)	14 (20.0)	2 (3.0)	7 (10.4)
19F	0	13 (36.1)	0	1 (1.4)	20 (30.3)	1 (1.5)
15B/C	4 (7.0)	6 (16.7)	4 (10.3)	5 (7.1)	8 (12.1)	8 (11.9)
11A	1 (1.8)	4 (11.1)	4 (10.3)	1 (1.4)	7 (10.6)	8 (11.9)
35F	5 (8.8)	2 (5.6)	0	5 (7.1)	5 (7.6)	1 (1.5)
19A	8 (14.0)	0	0	9 (12.9)	0	0
6B	1 (1.8)	0	0	1 (1.4)	6 (9.1)	1 (1.5)
14	0	2 (5.6)	2 (5.1)	0	5 (7.6)	3 (4.5)
22	0	5 (13.9)	0	0	7 (10.6)	0
Others	8 (14.0)	2 (5.6)	2 (5.1)	9 (12.9)	3 (4.5)	3 (4.5)
Non-typables	0	0	0	0	3 (4.5)	6 (9.0)

Total	**57(100%)**	**36 (100%)**	**39 (100%)**	**70 (100%)**	**66 (100%)**	**67 (100%)**

Distributions are presented separately for the index children and all individuals in the cohort in question.

Although carriage in the DCC's was concentrated in children, clustering of carriage was notable also in the total cohort of individuals linked to the DCC (index children, their family members and the day care employees). In fact, serotype 9V was still observed only in the Tommila cohort (36% of all 70 pneumococcal isolates found in this cohort). Serotype 18C was found only in the Salorinne cohort (43% of 67 isolates), and 20 of the 22 isolates of type 19F isolates were found in the Kuusimäki cohort (30% of 66 isolates).

By contrast, serotype 3 was present in all three DCCs during the study period, and it was the most common serotype in the whole data set of index children. It did not present in clear clusters as the other prevalent serotypes.: Moreover, it showed little spread to the employees of family members compared to the other common, more epidemically behaving serotypes.

### Family level

We assessed the dependence of serotype specific acquisition rates on exposure to pneumococci (Table 4). The acquisition rate of a new (target) serotype in the index children was 0.45/100 per child per month when there was no exposure to the target type neither in the family nor in the DCC. With exposure to carriage of the target type in the DCC but not in the family, the acquisition rate of a target type in the index children was 2.51/100 per index child per month. With exposure both in the family and the DCC, the rate was highest at 12.5/100 per month. The rate based on sole family exposure is also of interest, but as there was only one acquisition of pneumococcal carriage in the index children with exposure in the family but not in the DCC, it was difficult to infer its magnitude independently. By regression analysis, with the assumption of a multiplicative rate for carriage in both mixing groups, the relative rate was 5.3 (90% CI 2.9–10.0) for carriage in family only, and 5.4 (90% CI 3.6–8.2) for carriage in DCC only in comparison to "no observed carriage". For exposure in both mixing groups the relative rate was thus 28.8.

**Table 4:** The effect of exposure on the rate of pneumococcal acquisition in day care attendees.

*Exposure stratum*	Person-months* in children at risk of acquisition	*Number of acquisitions*	**Rate of acquisition per month per 100 children (90% confidence interval)**
None	7732	35	0.45 (0.34 – 0.60)
DCC only	1074	27	2.51 (1.83 – 3.45)
Family and	64	8	12.50 (7.00–22.32)
DCC			
Family only	22	1	4.55 (0.88–23.43)

Total	8892	71	0.80 (0.66 – 0.97)

Acquisition rates were determined in the following exposure strata: no observed carriage, carriage in the day care centre (DCC) only, carriage in both and carriage in the family only. The acquisition rates were calculated from the number of acquisitions for children at risk, i.e., not carrying the target type at the previous visit. The same acquisition rate was assumed for all serotypes.

* person-months were calculated from the number of children at risk at the start of the one-month observation interval, each contributing one month of person-time

Pneumococcal carriage was more prevalent in DCCs than families and, consequently, acquisition of carriage in the index child was clearly associated with previous carriage in the DCC. Of the 36 acquisitions with exposure to homologous pneumococci within family or DCC, the child had been exposed in the DCC in 35 cases (Table 4). In the remaining other 35 acquisitions, exposure had not been observed but may have occurred in DCC because of incomplete sampling. Based on the much lower rate of acquisition in this exposure class, however, it seems plausible that the amount unobserved exposure is not considerable and that most of these cases pertain to acquisition of carriage from the community.

The index child was the most likely route of introducing pneumococci to the family. There were 92 instances when a new serotype was introduced to a family (family acquisitions), and in 66% of these (61/92) the introductory individual was the index child. In 7 of these instances there was a co-introductory carrier in the family. When the index child was an introductory carrier, in 62% of the 61 cases the homologous serotype was found in the child's DCC at the same or the previous visit. Carriage in an index child increased the risk of acquisition in other family members 17-fold, compared to the baseline rate of 0.2 per 100 person-months when not exposed to carriage in the index child.

Of the 45 families involved, 6 had no pneumococcal carriage observed during the follow-up. The size of these families was not different from those with carriage, and the proportion of missing data was not significantly different from that in families with carriage.

### School data

In the cross-sectional study of carriage among school children, 9.4% (16/170) were positive for pneumococci, corresponding to the frequency of carriage among the siblings of the main study. Of the 16 pneumococcal isolates, 3 were of serotypes 18C and 10, and 2 isolates of 11A and 16. Serotypes 9V, 19F or 3 were not detected at all. There was one isolate of type 23F and one of type 6B. There was no obvious clustering of pneumococcal serotypes in the school classes, only 2 isolates of serotype 10 were found in the same class.

## Discussion

In this study we followed pneumococcal carriage in 59 day care attendees, their family members and the employees in three Finnish day care centres for 9 months. We showed that exposure to pneumococci both within day care centre (DCC) and within family are important risk factors for acquisition of carriage in children when measured with the same accuracy. Exposure confined to the DCC or family only signified a 5-fold rate of acquisition compared to no exposure in the family nor DCC. Simultaneous exposure both in DCC and in family further increased the relative rate to almost 30-fold. Pneumococcal carriage was more prevalent in DCCs than in families, and consequently the majority of acquisitions in the day care attendees was associated with carriage of the homologous type in the DCC. As a whole, pneumococcal carriage appeared highly clustered. Each of the three day care centres, together with the cohort of individuals linked to it, was dominated by a serotype of its own (19F, 18C, 9V).

We have formerly shown that attendance to day care, as a proxy for exposure, was not associated with risk of carriage in young children when simultaneous type-specific carriage by family members was known [10]. This lack of association was probably due to the non-specificity of day care attendance as a measure of exposure in comparison to explicit knowledge of simultaneous within-family carriage at the serotype level. The children in the earlier study were also younger and attended communal day care less than the children in the present one. The main motivation of the present study was to gather data about exposure to pneumococci on equal footing from families and day care. With these new data, we could indeed show that acquisition of carriage in children is strongly associated with previous carriage of homologous serotypes in both mixing groups.

Although exposure to pneumococci within families increased the risk of pneumococcal acquisition in the day care attendee, exposure was highly more prevalent in day care facilities than families. Consequently, acquisition of pneumococcal carriage by the day care attendee was strongly associated with previous exposure to a homologous type in the DCC: in the 36 acquisitions with known exposure within the family or the DCC, the child had been exposed in the DCC in 35 cases and in the family in 9 cases (Table 4).

The serotype distribution in this study clearly deviates from the expectation based on contemporaneous or preceding studies of pneumococcal carriage in the same location[10,22,23]. In general, most studies in Finland and elsewhere have revealed a surprisingly stable serotype distribution, with the same, so called paediatric serotypes appearing prevalent in young children (6B, 6A, 19F, 23F). Most strikingly, serotype 23F was completely absent in day care cohorts in our material and types 6A and 6B were rare. The skewed serotype distribution in the present study is due to the fact that we sampled day care cohorts rather than individuals. The observed pattern of carriage can be interpreted as manifestations of micro-epidemics, that is, clustering of serotype specific carriage in the mixing groups.

The almost complete absence of many common serotypes in our material actually strengthens its micro-epidemic interpretation, with low background (community) rates of acquisition for single serotypes and much higher within-group rates (see e.g.[24]). In particular, the fact that a follow-up of 59 children for 9 months did not result in a single observation of 23F carriage implies that the acquisition rate from the community even for this type is low. In fact, the rate of 0.0044 per month (Table 4) indicates a mean waiting time of 3.9 months until appearance of a new pneumococcal serotype in a cohort of 59 children, which is not in gross contradiction with the observed absence of 9 months for 23F.

In most family studies, the reported serotype distribution has resembled that from a random sample of individuals, the "common" serotypes being most prevalent [2,6,10,25,26] even though clear pneumococcal clusters have been seen in large families [27]. An obvious explanation is the fact that a family/household is too small a unit to exhibit extended micro-epidemics. By contrast, some previous studies based on day care centres have specifically reported clustering of pneumococcal carriage among children attending day care [28,29]. A larger group size and a bigger proportion of children, who are more prone to carry, allow pneumococcal microepidemics to be seen in studies reporting carriage in day care. In fact, school studies, especially cross sectional ones, with class size 20 and roughly 10% carriage, would be able to present only clusters of size 2 on average. This was actually seen also in our data (2 serotype 10 isolations in one class), which intuitively did not appear to show pneumococcal clusters.

The frequency of carriage in children was lower (mean 25%, range at the ten sampling rounds to the DCCs 14–44%) in our material than in many other published studies, taking into account that children attending day care are particularly assumed to carry pneumococcus more than children cared for at home. The day care attendees in our study were on average 4 years old, thus being older than in most of the studies concentrating on the first pneumococcal acquisition and hence sampled during the first 2 years of life, a period of heavier carriage. When comparing to other carriage data collected from the same location previously or simultaneously, the frequency of carriage in the present study is of the same magnitude: 25% vs. 28% [10] and 30% [23]although somewhat lower than expected as all children in the present study were attending day care.

There are obvious limitations to our study. First, a considerable proportion of day care attendees were not enrolled in the study and the information about exposure to pneumococci within day care is therefore not complete. With the assumption that sampling and transmission were not associated, however, the un-sampled group of day care attendees should have harboured similar serotypes as the sampled one. Moreover, the risk of acquiring pneumococcus was clearly lowest in the 'non-exposure' class (Table 4), implying that large amount of exposure cannot have remained hidden in the un-sampled children of the DCC. The strong clustering of carriage within DCC obviously means that most circulating serotypes were found.

Second, a study based on only three day care centres is inevitably small to fully characterize the micro-epidemic potential of particular pneumococcal serotypes. The serotypes identified as dominant in the present study, i.e., those found to cause micro-epidemics, are not necessarily the most transmissible ones. It is probable that e.g. serotypes 23F, 6A and 6B transmit in a similar fashion in the child population. In fact, we conjecture that practically all relatively common serotypes can form clusters. Therefore, a larger sample of DCCs would have presented clusters of the "usual" serotypes. Although serotype 3 appeared somewhat different, this study did not address differences across serotypes in transmissibility. It also remains a question whether micro-epidemic patterns are determined solely by heterogeneous transmission in a structured population of interconnected "patches" (day care cohorts), or if the dynamics of transmission needs to be augmented by competition in which one serotype hinders the presence, or at least compromises the sensitivity of finding others.

In conclusion, we hypothesize that pneumococci transmit in a population of "day care cohorts" and is regulated by the strength of within-cluster micro-epidemics. Specifically, because transmission is more intense within than across clusters, the ensuing micro-epidemic behaviour enhances pneumococcal transmission. This is similar to the role of core groups of disease transmission [30]. In a homogeneously mixing population, the vaccination effort to eradicate an SIS (susceptible-infectious-susceptible) type infection is typically of the order of the endemic prevalence of carriers. Due to within-cluster transmission, the critical efficacy for a vaccine to eradicate pneumococcal transmission may actually be larger than in a homogeneously mixing population with the same prevalence of carriage (cf. [24]). Microepidemic behaviour of carriage within host clusters may also have other bearings on pneumococcal epidemiology. For example, it is reflected in the genetic composition of the pneumococcal population, thus being an essential factor when attempting to elucidate the evolutionary mechanisms of pneumococci [31].

## Competing interests

The authors declare that they have no competing interests.

## Authors' contributions

TL participated in study design, coordination and analysis and was primarily responsible for writing the manuscript. FH performed data analysis and contributed to writing. RS participated in study design and coordination, sample collection and helped to draft the manuscript. AT was responsible for data design. KA participated in study design, carried out statistical analyses and was involved in writing the manuscript. All authors have read and approved the final manuscript.

## Pre-publication history

The pre-publication history for this paper can be accessed here:


